# A novel molecular and clinical staging model to predict survival for patients with esophageal squamous cell carcinoma

**DOI:** 10.18632/oncotarget.11362

**Published:** 2016-08-18

**Authors:** Wei Wang, Zhiwei Wang, Jun Zhao, Min Wei, Xinghua Zhu, Qi He, Tianlong Ling, Xiaoyan Chen, Ziang Cao, Yixin Zhang, Lei Liu, Minxin Shi

**Affiliations:** ^1^ Department of Surgery, The Affiliated Tumor Hospital of Nantong University, Nantong, Jiangsu Province, China; ^2^ Department of Breast, International Peace Maternity and Child Health Hospital, Shanghai Jiao Tong University, Shanghai, China; ^3^ Department of Thoracic Surgery, Shanghai Renji Hospital Affiliated to Shanghai Jiao Tong University School of Medicine, Shanghai, China; ^4^ Department of Thoracic and Cardiovascular Surgery, The First Affiliated Hospital of Soochow University, Medical College of Soochow University, Suzhou, Jiangsu Province, China; ^5^ Department of Pathology, The Affiliated Tumor Hospital of Nantong University, Nantong, Jiangsu Province, China; ^6^ Department of Pathology, Shanghai Ruijin Hospital Affiliated to Shanghai Jiao Tong University School of Medicine, Shanghai, China

**Keywords:** esophageal squamous cell carcinoma, UBE2C, MGP, survival

## Abstract

Current prognostic factors fail to accurately determine prognosis for patients with esophageal squamous cell carcinoma (ESCC) after surgery. Here, we constructed a survival prediction model for prognostication in patients with ESCC. Candidate molecular biomarkers were extracted from the Gene Expression Omnibus (GEO), and Cox regression analysis was performed to determine significant prognostic factors. The survival prediction model was constructed based on cluster and discriminant analyses in a training cohort (N=205), and validated in a test cohort (N=207). The survival prediction model consisting of two genes (UBE2C and MGP) and two clinicopathological factors (tumor stage and grade) was developed. This model could be used to accurately categorize patients into three groups in the test cohort. Both disease-free survival and overall survival differed among the diverse groups (P<0.05). In summary, we have developed and validated a predictive model that is based on two gene markers in conjunction with two clinicopathological variables, and which can accurately predict outcomes for ESCC patients after surgery.

## INTRODUCTION

Esophageal carcinoma ranks among the top ten most common malignancies in the world [1]. In China, more than 90% of esophageal cancer cases are esophageal squamous cell carcinoma (ESCC), which is the sixth most prevalent cancer in the country [2]. Despite recent improvements in surgical techniques and medical treatments, the overall prognosis for patients with ESCC remains poor [3–6]. Similarly as in other solid cancers, TNM staging has been widely used to estimate survival and make clinical decisions about adjuvant therapy for ESCC patients [7]. Indeed, this staging system has improved survival of ESCC patients in the past. However, current staging methods and therapeutic decisions remain suboptimal.

ESCC patients with the same stage and similar treatment may have quite different clinical outcomes. Importantly, patients with early-stage cancer and low risk of recurrence could be spared the toxicity of systemic treatment if clearly distinguished, while others at high risk of distant recurrence could get maximal benefit from adjuvant therapy. It is increasingly recognized that a tremendous heterogeneity between patients exists in the biology underlying ESCC. Hence, the ideal staging system would take into account the biology and molecular features of each individual tumor and correlate prognosis with patient-specific tumor biomarkers.

Recently, gene expression profiles enabled to predict outcomes and select patients for adjuvant therapies in colon cancer, lung cancer, hepatocellular carcinoma, breast cancer, and esophageal adenocarcinoma [8–13]. Unfortunately, practical predictive models for the outcomes of patients with ESCC after curative resection are not currently available. In this study, we investigated the prognostic value of the traditional clinicopathological factors and selected protein expression in a training cohort. We aimed to develop a novel predictive model, which would be capable to predict outcomes of ESCC patients after surgery.

## RESULTS

### Candidate gene selection

During data analysis, we found that 8171 genes were differentially expressed. Among these genes, 3972 genes were up-regulated and 4199 genes were down-regulated. From 75 microarray samples, 115 genes were identified, and 8 genes were randomly selected for further analysis: MGP, SPP1, SULF1, NECAB1, SPARC, GLIPR1, UBE2C and KCNB1 (Supplementary Table S1).

### Patients and follow-up

Patients' follow-up was performed until the end of September 2014. The median follow-up time was 48 months (range 2 to 105 months) in the training cohort and 50 months (range 2 to 93 months) in the validation cohort. During the follow-up period, a total of 153 all-cause deaths were observed in the training cohort and 115 in the validation cohort. 160 patients had recurrence or metastasis in the training cohort and 140 in the validation cohort. No differences in the distributions of basic characteristics were observed between training and validation cohorts (P>0.05) (Table 1).

**Table 1:** Characteristics of study participants in the training and validation datasets

Variables	Training (n=205)		Validation (n=207)		P-value
	No.	%	No.	%	
Age (years)					0.107
<60	78	38.0	95	45.1	
≥60	127	62.0	112	54.9	
Gender					0.263
Male	149	72.7	140	67.6	
Female	56	27.3	67	32.4	
Tumor size					0.139
<5cm	93	45.4	109	52.7	
≥5cm	112	54.6	98	47.3	
T stage					0.311
pT1	31	15.1	43	20.8	
pT2	55	26.8	61	29.5	
pT3	104	50.7	88	42.5	
pT4	15	7.3	15	7.2	
Lymph nodes status					0.084
Negative	126	61.5	144	69.6	
Positive	79	38.5	63	30.4	
TNM stage					0.183
I	53	25.9	72	34.1	
II	68	33.1	63	29.9	
III	84	41.0	76	36.0	
Grade					0.750
Well-differentiated	30	14.6	34	16.4	
Moderately-differentiated	102	49.8	106	51.2	
Poorly-differentiated	73	35.6	67	32.4	
Vascular invasion					0.505
Positive	16	7.8	20	9.7	
Negative	189	92.2	187	90.3	

### Gene expression analysis

**Figure 1:**
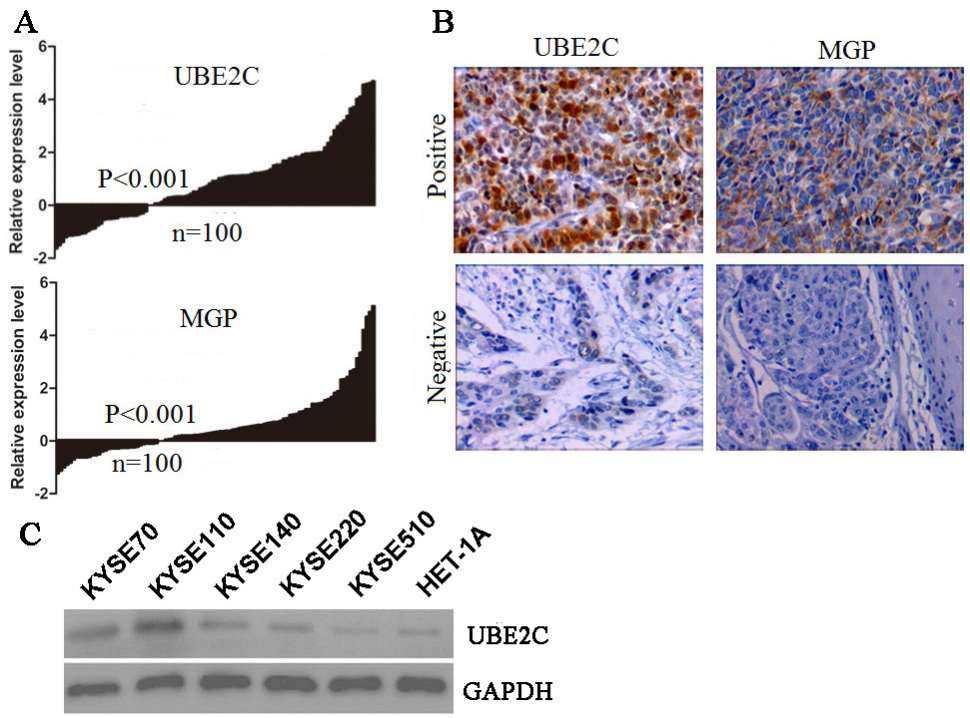
The candidate gene expression in the training cohort and cell lines **A.** Quantitative RT-PCR of two selected genes, UBE2C and MGP. **B.** Representative IHC staining showing protein expression in the invasive tumors (×200). **C.** The western blot analysis of UBE2C protein in five ESCC cell lines (KYSE) and human normal esophageal squamous epithelial cell line (Het-1A).

Quantitative RT-PCR was carried out to analyze expression of the eight selected genes in 100 tumor and non-tumor tissues. Consistent with the results from microarray data analysis, two of the eight genes, UBE2C and MGP, were highly expressed in tumor tissues (Figure 1A). However, there was no significant difference between tumor and non-tumor tissues in the mRNA levels of the other six genes (SPP1, SULF1, NECAB1, SPARC, GLIPR1 and KCNB1) (Supplementary Figure S1). Hence, UBE2C and MGP genes were selected for survival model training.

### UBE2C and MGP protein expression analysis

To evaluate the potential use of UBE2C and MGP as biomarkers for ESCC, we analyzed the protein levels of UBE2C and MGP in 205 training ESCC tumor tissues by immunohistochemistry (IHC). For both proteins examined, we scored nuclear, cytoplasmic, and membrane staining. In the carcinoma cells, UBE2C was found both in the nuclei and in the cytoplasm. In contrast, MGP was found exclusively in the cytoplasm in the carcinoma cells. The positive rates of UBE2C and MGP in ESCC tumor tissues were up to 43.4% (89/205) and 41.5% (85/205), respectively (Figure 1B). The UBE2C protein expression levels of five human ESCC cell lines (KYSE-70, KYSE-110, KYSE-140, KYSE-220, KYSE-510) and a human normal esophageal squamous epithelial cell line (Het-1A) were assessed by western blot (Figure 1C). The KYSE-70, KYSE-110, KYSE-140 and KYSE-220 ESCC cells showed high-UBE2C expression, as compared to normal epithelial cell with low UBE2C expression.

### Construction of survival prediction model for training cohort

**Figure 2:**
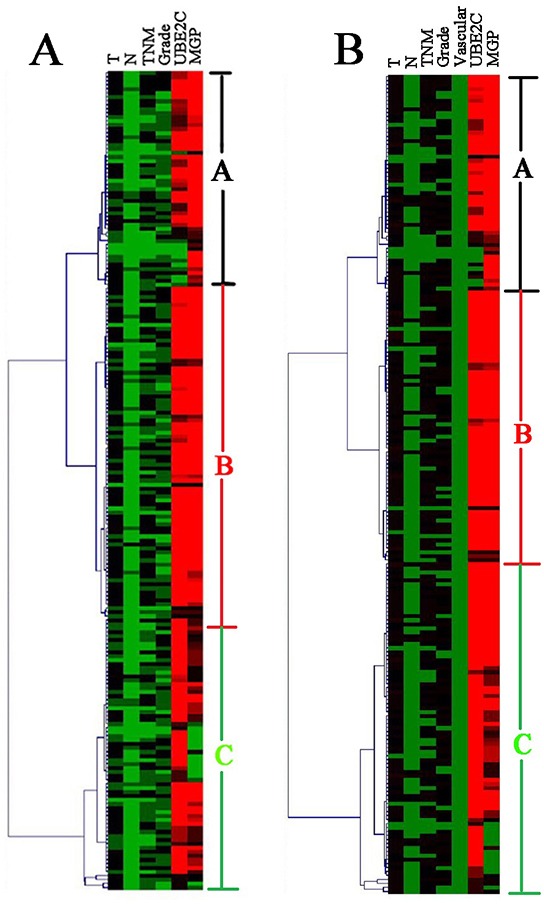
Unsupervised cluster analysis of the training set of 205 ESCC genes identified three clinically relevant subsets (group A, B and C) Each row is a sample, and each column is a prognostic factor. High value is depicted as red, low value as green and median value as black. **A.** The clustering results by the prognostic factors for disease-free survival. **B.** The clustering results by the prognostic factors for overall survival.

For training cases, univariate analysis showed that the factors of T stage, lymph nodes status, TNM stage, tumor grade, UBE2C expression and MGP expression were significantly associated with disease-free survival (DFS) (P<0.05) (Table 2). These prognostic factors were used to perform unsupervised hierarchical clustering analysis. The tree-view program displays the data with the prognostic factors on the horizontal axis and the cases on the vertical axis. The cases and prognostic factors are arranged in such a way that the cases with the most similar expression profiles are placed next to each other. The length of the dendrogram branches connecting the cases or prognostic factors, is inversely proportional to the similarity of their profiles. Clustering of 205 training ESCC cases produced three groups, groups A, B, and C, which can be discerned from the dendrogram (Figure 2A). Groups A, B and C contained 52, 85 and 68 cases, respectively.

**Table 2:** Univariate Cox proportional hazards regression for disease-free survival and overall survival in training cohort

Variables	Category	Disease-free survival			Overall survival		
		HR	95%CI	P-value	HR	95%CI	P-value
T stage	pT1+ pT2	1.00			1.00		
	pT3+ pT4	1.82	1.30-2.55	<0.001	1.86	1.33-2.60	<0.001
Lymph nodes status	N0	1.00			1.00		
	N1	1.75	1.32-2.16		1.80	1.29-2.31	
	N2	2.18	1.63-2.72		2.62	2.13-3.10	
	N3	2.43	1.98-2.89	<0.001	2.94	2.27-369	<0.001
TNM stage	I	1.00			1.00		
	II	1.56	1.30-1.82		1.61	1.42-1.86	
	III	2.18	1.74-2.62	<0.001	2.33	1.78-2.68	<0.001
Grade	Well-differentiated	1.00			1.00		
	Moderately-differentiated	1.33	0.85-2.07		1.34	0.86-2.09	
	Poorly-differentiated	2.23	1.39-3.58	0.001	2.30	1.43-3.69	0.001
Vascular invasion	No	1.00			1.00		
	Yes	1.67	0.98-2.81	0.057	1.71	1.02-2.92	0.046
UBE2C	Negative	1.00			1.00		
	Positive	2.50	1.81-3.46	<0.001	2.55	1.84-3.52	<0.001
MGP	Negative	1.00			1.00		
	Positive	1.95	1.42-2.67	<0.001	1.96	1.43-2.68	<0.001

**Figure 3:**
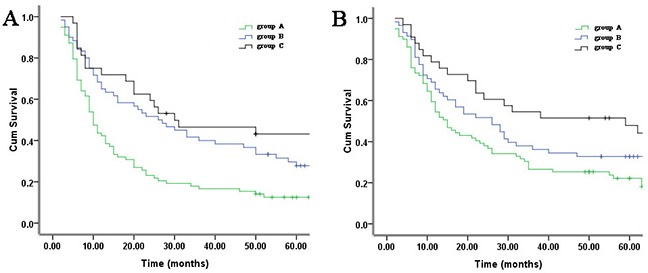
Kaplan-Meier analysis of three clusters of 205 training set identified by hierarchical clustering (groups A, B, C) Differences in survival between subgroups are assessed by log-rank tests. **A.** Disease-free survival (P <0.001). **B.** Overall survival (P <0.001).

Next, discriminant analysis was used to establish the survival prediction model by clustering results and to evaluate the efficiency of classification. The model was obtained as follows:

Y1=5.400T-5.763N+6.115TNM+4.066Grade+0.01UBE2C+0.024MGP-14.340;

Y2=4.334T-4.804N+5.726TNM+4.205Grade-0.026UBE2C+0.034MGP-12.399;

Y3=5.078T-4.984N+5.964TNM+3.869Grade+0.026UBE2C-0.024MGP-13.085;

Y(1,2,3) is the probability of groups A, B and C. Classifying rule: if Y1> Y2 and Y3, group A case; if Y2> Y1 and Y3, group B case; if Y3> Y1 and Y2, group C case. Correct classification rate reached 84.9%. Using the results from discriminant analysis, Kaplan–Meier curves showed that group C cases had a better DFS, whereas group A cases had a worse DFS. The 5-year DFS rate for groups A, B and C patients was 17.9%, 31.5% and 43.2%, respectively (P <0.001) (Figure 3A).

The Cox regression analysis showed that T stage, lymph nodes status, TNM stage, tumor grade, vascular invasion, UBE2C expression and MGP expression were significantly associated with overall survival (OS) (p<0.05) (Table 2). These prognostic factors were used to perform unsupervised hierarchical clustering analysis. Two hundred and seven training cases were divided into three groups. Groups A, B and C included 53, 69 and 83 cases, respectively (Figure 2B). Discriminant analysis showed the prediction model for OS as follows:

Y1=4.326T-4.608N+6.007 TNM+4.058Grade-0.025UBE2C+0.034MGP-12.468;

Y2=5.138T-5.077N+5.671TNM+4.001Grade+0.026UBE2C-0.025MGP-12.955;

Y3=5.379T-5.394N+6.497TNM+3.839Grade+0.004UBE2C+0.019MGP-14.312;

Correct classification rate reached 82.9%. Using the results from discriminant analysis, the survival results showed that group C cases had a better OS, whereas group A cases had a worse OS. The 5-year OS rate for groups A, B and C patients was 22.2%, 37.5% and 47.8%, respectively (P <0.001) (Figure 3B).

### Survival prediction for testing patients

**Figure 4:**
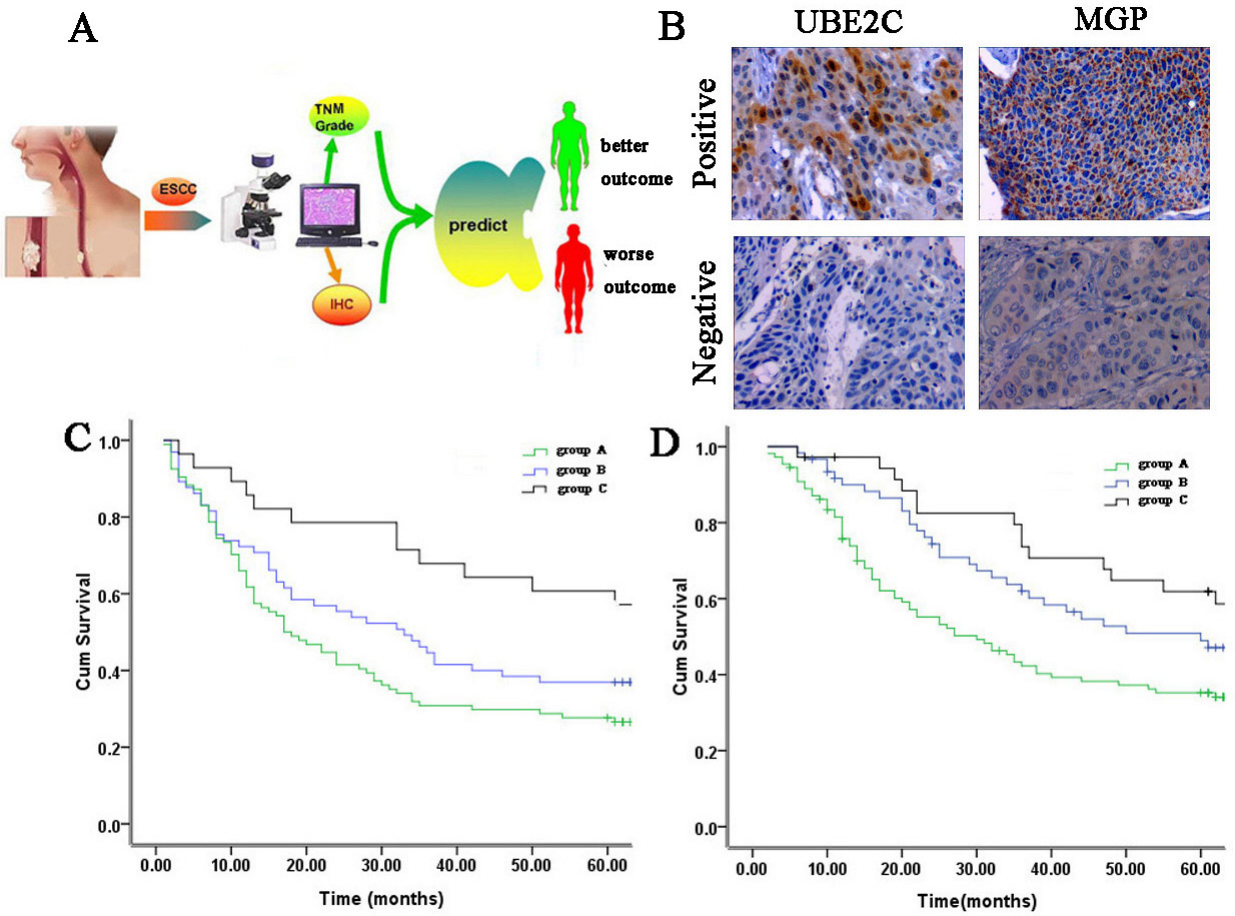
Survival prediction in the testing cohort **A.** Schematic layout of survival prediction. **B.** Representative IHC staining showing protein expression in the invasive tumors in testing set (×200). **C.** Postoperative survival curves of disease-free survival in the testing cohort based on the survival prediction model by the Kaplan-Meier analysis and log-rank test (P=0.006). **D.** Postoperative survival curves of overall survival in the testing cohort based on the survival prediction model by the Kaplan-Meier analysis and log-rank test (P=0.002).

The survival prediction model was applied to an independent test group of 207 ESCC patients (Figure 4A). The positive rates of UBE2C and MGP in ESCC tumor tissues were up to 56.5% (117/207) and 53.6% (111/207), respectively (Figure 4B). For DFS, 65, 94 and 48 cases were classified as groups A, B and C respectively. As shown in Figure 4C, group C cases had a significantly better DFS, whereas group A cases had a significantly worse DFS. The 5-year DFS rate for groups A, B and C patients was 28.7%, 38.5% and 57.1%, respectively(P=0.00 6). With regard to overall survival, the predictive results showed that 55, 99 and 53 cases were classified as groups A, B and C respectively. As shown in Figure 4D, there was a significant difference in OS among diverse groups. The 5-year OS rate for groups A, B and C patients was 32.1%, 47.1% and 58.6%, respectively (P=0.002).

## DISCUSSION

The clinical outcomes of ESCC are heterogeneous, and the available prognostic markers are limited. Currently, only the stage based on TNM classification is widely accepted as a prognostic factor, which is still far from an accurate prediction. To improve prognosis, we hypothesized that a combination of clinical features and well-validated molecular markers would allow stratification of ESCC patients into clinically meaningful prognostic subgroups. In our study, eight genes were initially selected from microarrays. After confirming their increased expression in tumor tissues by quantitative RT-PCR, two of the eight genes were included in constructing the survival prediction model. Finally, a predictive model containing two proteins and traditional staging was developed in a training cohort. Examination of training samples resulted in a predictive accuracy of more than 80%. Both training cases and testing cases had a worse survival. It is possible that higher tumor stage and overexpression of UBE2C and MGP in group A have contributed to the observed trend of worse outcome, because both factors have been associated with poor survival in ESCC.

Ubiquitin conjugating enzyme E2 C, UBE2C, is required for degradation of mitotic cyclins, regulation of anaphase-promoting complex, and for cell cycle progression [14, 15]. Overexpression of UBE2C causes chromosome missegregation and alters the cell cycle profile, thus facilitating cell proliferation [16, 17]. Increased expression of UBE2C has been observed in various solid cancers including breast cancer, non-small cell lung cancer, bladder cancer, and ovarian cancer [18–22] As for ESCC, a recently published study has indicated that elevated level of UBE2C is associated with a more advanced clinical stage and worse prognosis [23]. We have discovered that UBE2C was up-regulated in group A, which was characterized by worse outcome. In esophageal adenocarcinoma (EA), the expression of UBE2C was elevated compared to Barrett's metaplasia, and was suppressed by proteasome inhibition, resulting in suppressed cell proliferation and cell cycle [24].

Matrix Gla protein, MGP, is a calcium-binding and vitamin K-dependent protein, which is found in bone matrix and cartilage [25]. The MGP gene is overexpressed in different types of cancer, including ovarian, lung, urogenital, skin, and glioblastomas [26–30]. In glioblastoma, MGP promotes tumor growth and angiogenesis [31]. Up-regulation of MGP mRNA correlates with poor prognosis for breast cancer and gastric cancer [32, 33]. However, conflicting findings exist in colorectal cancer, where MGP mRNA does not seem to correlate with histopathologic features, such as tumor progression, size and cell differentiation [34]. Up to now, there was no study on MGP in ESCC. Our study has found that ESCC patients with higher MGP expression in group A have poor clinical outcome.

Previous studies have used expression profiles to predict prognosis in EC, where overall survival is the main outcome indicator [35–37]. In our study, not only overall survival but also disease-free survival were considered as end points. Although tumor recurrence and overall survival are closely related, it is not uncommon for some patients with localized recurrence to benefit from radical treatment resulting in relatively prolonged survival. Furthermore, the overall endpoint is affected by competing risks due to comorbidities. We considered prediction of tumor recurrence as a superior end point to overall survival for our purpose of developing survival predictive model to identify patients who would benefit from adjuvant treatment.

Several biases can be associated with the patient cohort included in this study. The patients included in our study were treated at two hospitals for several years. The training cohort has a relative overrepresentation of late progressing tumors compared with the test cohort. This may complicate a comparison of the training cohort with the validation cohort. Therefore, a multicenter study under standardized conditions needs to be performed before a possible clinical use. In addition, although IHC can be easily performed in standard clinical pathology laboratories, the IHC scoring is subjective; however, this problem can be minimized using standard-intensity pictures to allow for accurate classification of the staining pattern.

In summary, this study demonstrates that the survival of ESCC patients can be predicted with a reasonable accuracy by using a simple discriminant function based on UBE2C and MGP gene expression and clinical stage. Our methodology may be important to both patients and physicians in terms of life planning and therapeutic strategies.

## MATERIALS AND METHODS

### Microarray data analysis

Publicly available microarray data in Gene Expression Omnibus (GEO, under the accession number GSE 13898) were used to extract the candidate genes. R/BioConductor [38] was used for processing the microarray text data from BeadStudio. The expression level of each gene was transformed into a log 2 base before further analysis. The log 2 fold change (FC) of each probe on the array within each tissue pair was then calculated. Rank product testing was performed to test whether the differential expression between tumor tissue and matched normal mucosa was significant. The differential expression was considered significant if the adjusted p-value, i.e. the FDR q-value, was less than 0.05.

### Study population

A total of 412 patients who had undergone esophagectomy for ESCC at Nantong tumor hospital (between January 2007 and July 2008) and Renji hospital, Shanghai (between January 2006 and September 2008) were retrospectively enrolled. ESCC was confirmed by postoperative histologic pathology in all cases. The recruited cases met the following eligibility criteria: a minimum of 50% tumor cells present in the tissue section, R0 resection, no previous malignancy. Resected tumor and paired non-tumor tissue specimens were immediately cut from the resected esophagus and placed in RNA-Later (TaKaRa, Japan), frozen in liquid nitrogen and kept at −80°C until RNA extraction. One hundred and twenty-two patients received adjuvant chemotherapy and eighty-four patients received adjuvant radiotherapy (50 Gy, 2 Gy/day for five days/week for five weeks) after surgery. The most common chemotherapy regimen consisted of paclitaxel plus cisplatin for a mean of 3 cycles after surgery, depending on clinical response or the occurrence of adverse effect.

This study was approved by the institutional review board and ethics committee at Nantong tumor hospital and Renji hospital. The written informed consents were obtained from all patients.

### Patient follow-up

Uniform follow-up protocol was carried out at two centers. The patients were examined every 3 months for the first 2 years after operation, every 6 months for the following 3 years, and yearly thereafter. After being discharged from the hospital, recording of medical history, physical examination, endoscopic biopsy, CT, and PET were performed during the follow-up time. The endpoints included disease free survival and overall survival. The disease free survival was defined as the interval from the date of surgery to the date of local or regional disease recurrence, distant metastasis, or to the last follow-up date. The overall survival was calculated from the time of surgery to the time of death from any cause, or to the time of last follow-up. Some patients received palliative treatment to improve their quality of life during disease recurrence. When local or regional disease recurrence was observed, patients were treated with chemoradiotherapy or chemotherapy. If distant metastasis was found, chemotherapy or best supportive care were given.

### Quantitative real-time PCR (qRT-PCR) and western blot

2 mg of mRNA from each sample was reverse transcribed to single stranded cDNA using an Advantage RT for PCR kit (Clontech). Gene expression was assessed by qRT-PCR using an Applied Biosystems 7500 Fast Sequence Detection System (Life Technologies Corp, CA, USA). The PCR reaction mixture consisted of QuantiTect SYBR Green PCR master mix (2X QuantiTect SYBR Green kit, which contains HotStart Taq® DNA polymerase, QuantiTect SYBR Green PCR buffer, dNTP mix, SYGB I, Rox passive reference dye and 5 mM MgCl_2_) (Qiagen), 0.5 μmol/l of each primer and cDNA. Glyceraldehyde-3-phosphate dehydrogenase (GAPDH) gene was used as an endogenous control to normalize the expression data. The primers used for qRT-PCR analysis are summarized in Supplementary Table S2. The comparative Ct (threshold cycle) method was used to calculate the relative changes in gene expression.

The five human esophageal squamous carcinoma cell lines (KYSE-70, KYSE-110, KYSE-140, KYSE-220, KYSE-510) and a human normal esophageal squamous epithelial cell line (Het-1A) were purchased from the DSMZ-German Collection of Microorganisms and Cell Cultures (Braunschweig, Germany). The cells were cultured in RPMI 1640 supplemented with 10% fetal bovine serum (FBS) at 37°C in a humidified atmosphere with 5% CO2. Total proteins from cells were extracted using RIPA buffer (Solarbio, Beijing, China) and the protein concentration was measured by BCA Protein Assay Kit (Pierce, Rockford, USA). Western blot was performed according to the standard protocol, with primary antibody against UBE2C (1:1000, Abcam) GAPDH was used as a control to confirm equal loading of protein. Tannon 2500 imaging system was used to analyze the band intensities.

### Immunohistochemical analysis

Paraffin-embedded samples (4 mm thickness) of ESCC tumors were used for histopathological analysis after antigen retrieval. Briefly, endogenous peroxidase was blocked by incubating the sections in a 0.3% solution of hydrogen peroxide (in PBS) for 10 min. Antigen retrieval was performed by heating the sections for 10 min at 100°C in 10 mM citrate buffer (pH 6.0). Sections were incubated overnight at 4°C with rabbit anti-UBE2C (1:300 dilution, ab12290, Abcam, Cambridge, UK) and mouse anti-MGP (1:50 dilution, SC-81546, Santa Cruz, Texas, USA) antibodies.

Immunostaining scoring was performed by using the semiquantitative H-score method to calculate the sum of the percentage and intensity of positively stained invasive tumor cells. The staining intensity was divided into four grades: 0=negative staining; 1=weak staining; 2=moderate staining; 3= strong staining. Staining score was determined by the following formula: H score = percentage score x intensity score ((1*%1+)+(2 *%2+)+(3*%3+)), which ranged from 0 to 300 [39]. The X-tile software (version 3.6.1) was used to determine H-score cutoffs by dichotomizing patients according to H-score value and clinical outcome [40]. Positive staining was interpreted as H-score>45 for UBE2C and >50 for MGP. The cut points for each protein were defined in the Shanghai patient cohort (training set) and directly applied to Nantong patient cohort (validation set).

### Construction of survival prediction model and statistical analysis

**Figure 5:**
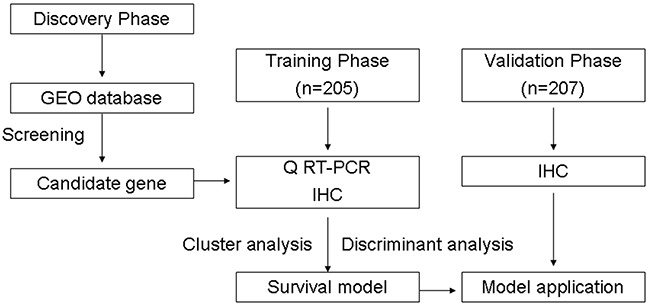
Study design

The prognostic significance of clinical and pathologic characteristics was determined using Cox regression analysis. The significant prognostic factors for training samples were selected for establishing survival prediction model. All training samples were categorized by unsupervised cluster analysis (Multi Experiment Viewer, MEV4-6-1). Following log transformation and centre to median calculations, average hierarchical clustering was performed using the Spearman rank correlation. We assigned one of three categories to cluster analysis data for each sample. Then, the Categorical data and significant prognostic factors were applied to construct survival prediction model by Bayer's discriminant analysis. The significance of discriminating variables was estimated using one-factor ANOVA F statistics. The set of optimal features was constructed using stepwise selection of variables minimizing overall Wilk's lambda statistic. Finally, we applied this prediction model to test a group of 205 samples. For the sample testing, each predictor was assigned one of three categories as those obtained in the training group. Figure 5 outlines the experimental flow of the study.

### Statistical analysis

The Kaplan-Meier method was used to estimate the survival of patients in different groups, and the two-side log-rank test was used to determine the statistical significance. Categorical data were presented as counts and group comparisons were made with the chi-squared test or the Fisher's exact test. All data were processed using SPSS 15.0 software package. The P values less than 0.05 were considered as significant.

## SUPPLEMENTARY FIGURE AND TABLES


