# Supplementary material for: A novel molecular and clinical staging model to predict survival for patients with esophageal squamous cell carcinoma
Source: Oncotarget. 2016 Aug 18;7(39):63526–36. doi: 10.18632/oncotarget.11362 (PMC5325382; doi:10.18632/oncotarget.11362)
Supplement: Supplementary file 1 [file oncotarget-07-63526-s001.pdf]

A novel molecular and clinical staging model to predict survival for patients with esophageal squamous cell carcinoma

SUPPLEMENTARY FIGURE AND TABLES

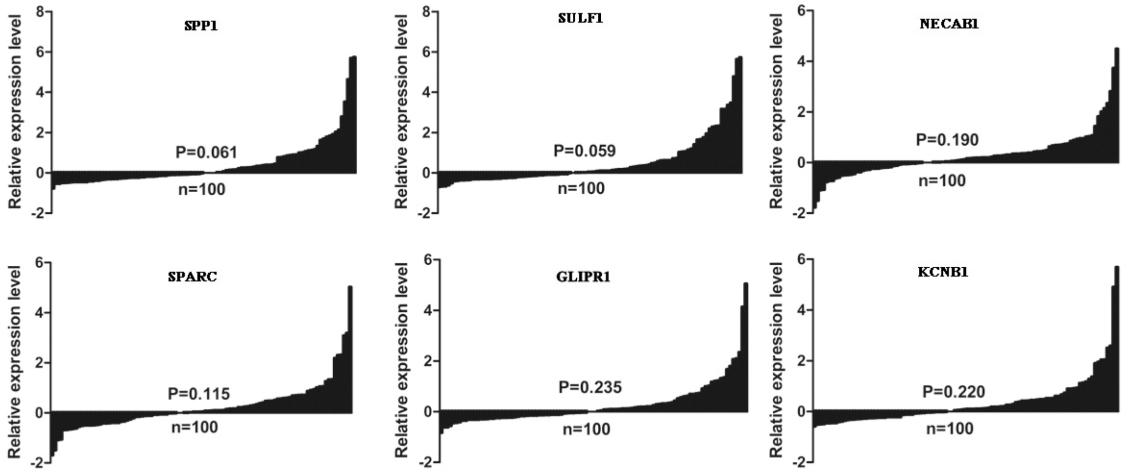

Supplementary Figure S1: Quantitative RT-PCR of six selected genes.

Supplementary Table S1: Lists of genes selected to validate in this study

Symbol	Gene title	P-value	Fold
MGP	matrix Gla protein	5.36E-07	10.026
SPP1	secreted phosphoprotein 1	2.83E-06	8.863
SULF1	sulfatase 1	4.93E-06	8.482
NECAB1	N-terminal EF-hand calcium binding protein 1	9.20E-05	6.516
SPARC	secreted protein, acidic, cysteine-rich (osteonectin)	1.60E-04	6.153
GLIPR1	GLI pathogenesis-related 1	4.36E-04	5.498
UBE2C	ubiquitin-conjugating enzyme E2C	2.07E-03	4.494
KCNB1	potassium voltage-gated channel, Shab-related subfamily, member 1	2.56E-03	4.358

Supplementary Table S2: qPCR primers used in this study

Gene	Primer sequences (5' -> 3')	Amplicon size
MGP	F'-AGTCCAAGAGAGGATCCGAG	100bp
	R'-ATAAACCATGGCGTAGCGTT	
SPP1	F'-CTCCATTGACTCGAACGACTC	230bp
	R'-CAGGTCTGCGAAACTTCTTAGAT	
SULF1	F'-GATCCCCGAGGTTCAGAGGA	178bp
	R'-GGTGTAGTCACAAAGGCATTGA	
NECAB1	F'-TCCAAGGGCATGTCGATCTTC	111bp
	R'-ACTGAGAACACCATCTGCAAAA	
SPARC	F'-TGAGGTATCTGTGGGAGCTAATC	128bp
	R'-CCTTGCCGTGTTTGCAGTG	
GLIPR1	F'-ACCCAAACTTCACTTCACTGG	102bp
	R'-AGTCCTGGATTTCGTCATACCA	
UBE2C	F'-GTCTGGCGATAAAGGGATT	178bp
	R'-ATAGCAGGGCGTGAGGA	
KCNB1	F'-ACTCTGGCGTACCCTGGAC	109bp
	R'-GTCGTCGAGGCTGTAGTCATC	
GAPDH	F'-GACATCAAGAAGGTGGTGAA	155bp
	R'-TGTCATACCAGGAAATGAGC	
